# Probing Surface Degradation Pathways of Charged Nickel-Oxide Cathode Materials Using Machine-Learning Interatomic Potentials

**DOI:** 10.1021/acsami.5c11818

**Published:** 2025-09-25

**Authors:** Svenja Both, Andrey D. Poletayev, Timo Danner, Arnulf Latz, M. Saiful Islam

**Affiliations:** † German Aerospace Center, Institute of Engineering Thermodynamics, Ulm 89081, Germany; ‡ Helmholtz-Institute Ulm for Electrochemical Energy Storage, Ulm 89081, Germany; § Department of Materials, 6396 University of Oxford, Oxford OX1 3PH, U.K.; ∥ The Faraday Institution, Harwell Science and Innovation Campus, Didcot OX11 0RA, U.K.; ⊥ Institute of Electrochemistry, Ulm University, Ulm 89081, Germany

**Keywords:** Li-ion batteries, Ni-rich cathode materials, surface degradation, density-functional theory, machine-learning interatomic potentials

## Abstract

While nickel-based layered oxide cathodes offer promising energy and power densities in lithium-ion batteries, they suffer from instability when fully delithiated upon charge. Ex situ studies often report a structural degradation of the charged cathode materials, but the precise mechanism is still poorly understood on the atomic scale. In this work, we combine high-level ab initio calculations with molecular dynamics using machine-learning interatomic potentials to study structural degradation of fully delithiated LiNiO_2_ surfaces at the top of charge. We find a previously unreported, stable reconstruction of the (012) facet with more facile oxygen loss compared to the pristine surfaces. The oxygen vacancy formation energy closely corresponds to the experimental decomposition temperatures of charged cathodes. Furthermore, we use molecular dynamics simulations to sample Ni ion migration into alkali-layer sites that is a kinetically plausible initiation step for surface degradation toward thermodynamically stable products.

## Graphical abstract

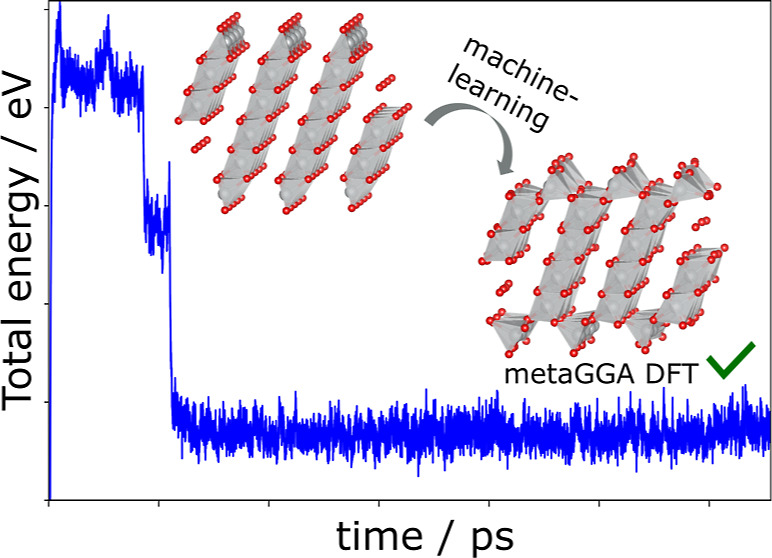

## 1 Introduction

Understanding degradation pathways in lithium-ion batteries is crucial to extending battery lifetimes. Ni-rich oxide cathodes have gained increasing importance^1^ since higher Ni content in, for example, the Li­(Ni,Mn,Co)­O_2_ series leads to higher capacities for given voltage cutoffs. Increasing capacity with Ni content corresponds to an increasing extent of reversible delithiation before decomposition sets in.^2^ Multimodal characterization points to a degradation process that includes the release of lattice oxygen with the formation of reduced and resistive oxide phases.^2–8^ While the evolved gases are usually measured using online electrochemical mass spectroscopy,^2,9,10^ the solid products can be studied using high-resolution imaging techniques such as transmission electron microscopy.^5,7^ The resulting solids comprise a mixture of spinel- and/or rocksalt-like phases,^5,7,8,11^ but their exact composition and structure remain unclear with reported products including NiO, Ni_3_O_4_, or LiNi_2_O_4_.^8,11,12^ In part due to the slow nature of cycling experiments, the thermal stability of charged cathodes can be used as a proxy for their stability in electrolytes.^3,4,8,13^ However, the nature and sequence of structural degradation pathways are not fully understood, especially on the atomic scale.

To complement and inform experiments, computational studies of delithiated cathodes focus on degradation pathways, including both the thermodynamics and kinetics of possible reaction mechanisms. The phase transformation that decomposes layered NiO_2_ into reduced metal-rich oxides and molecular O_2_ is thermodynamically favorable^14^ but has been predicted to be kinetically slow in the absence of defects.^15,16^ Therefore, the evolution of molecular O_2_ from Li-stoichiometric cathodes has been associated exclusively with a surface- or defect-initiated process. Li et al.^17^ studied the thermodynamics of the (104) and (001) surface terminations of LiNiO_2_ using static density-functional theory (DFT) calculations, suggesting an irreversible phase change upon delithiation. Kong et al.^15^ further suggested that Ni migration and oxygen dimerization could occur across the layers of the (104) surface of Li_0.28_NiO_2_ based on ab initio molecular dynamics (AIMD).

The (012) surface was predicted to change oxygen coverage in response to variation in the chemical potential of oxygen,^18,19^ which could affect its stability. Previous studies suggest that O_2_ evolution is spontaneous from the (012) surface of fully delithiated NiO_2_ with singly coordinated oxygen, i.e., under oxygen-rich conditions,^20–22^ but did not probe the loss of multiply coordinated oxygen, transition metal migration, or phase transformation.^20,21^ Additionally, most previous DFT studies of the stability of LiNiO_2_ have been performed at the generalized gradient approximation (GGA) level of theory using the Perdew–Burke–Ernzerhof (PBE) functional and a Hubbard *U* correction. However, this level of theory may not accurately represent the behavior of Ni-based layered oxides at low lithium content, as evidenced by its failure to reproduce the lattice contraction in Li_*x*_NiO_2_ upon delithiation to *x* < 0.25 ^23–25^ and the need to vary the Hubbard *U* parameter by more than 1 eV to reproduce the charging voltages of LiNiO_2_.^26^ More broadly, the under-stabilization of oxides in PBE remains an important source of possible systemic errors in studies of surface reactivity.^27^ Without the need for empirical parametrization with +*U*, metaGGA functionals such as SCAN predict the lattice contraction correctly^23^ and also reproduce oxide thermodynamics.^27,28^ More recently, r^2^SCAN^29^ has been shown to be broadly applicable^30^ and was successfully applied to bulk LiNiO_2_, capturing many physical properties of the material,^31^ including lattice contraction, thermodynamics, and Ni speciation across all states of charge.^31,32^

While metaGGA DFT enables an accurate representation of the underlying energetics, ab initio MD simulations at that level of theory have been too time-consuming to sample rare reactive events such as decomposition or transition metal migration. To overcome this limitation, machine-learning interatomic potentials (MLIP) have been proposed that enable long-time scales and large structures in reasonable simulation time.^33,34^ The CHGNet code used in this work is charge-informed by including on-site magnetic moments^33^ and has been used to run MD simulations of electrode materials including LiMnO_2_, LiFePO_4_,^33^ and Na-rich cathodes^35^ across various states of charge.

In this work, we combine metaGGA DFT calculations with ML-MD to study surface degradation at different surface facets of delithiated NiO_2_ cathodes. We reveal a novel surface degradation pathway and show that the formation of oxygen vacancies can trigger subsequent Ni migration, which is a key step toward forming stable, thermodynamic product phases. An important and novel point of consistency with experiments is the close correspondence of our calculated formation energies for surface oxygen vacancies with temperatures at which charged cathodes decompose thermally by oxygen evolution.

## 2 Results and Discussion

### 2.1 Structures and Energies of NiO_2_ Surfaces

We first focus on the fully delithiated O3–NiO_2_ material (Delmas notation^36^) since oxygen release and phase transformation are known to occur at very low states of lithiation during and after a two-phase delithiation from Li_0.25_NiO_2_.^2,37^ Figure 1a shows the calculated surface energies for various crystal facets of fully delithiated NiO_2_, including the (104), (012), (001), and (110) facets (calculated according to eq 1 in the Methods section). We use a symmetric oxygen-stoichiometric (012) surface with half-monolayer coverage of oxygen in order to maintain a dipole-free slab^18^ and avoid oxygen-rich terminations for the otherwise polar (012) surface, which are thermodynamically unstable under most conditions (Supporting Information). The (001) facet of NiO_2_ is the most stable with a surface energy of 0.23 J m^–2^, consistent with a full coordination of the NiO_6_ octahedra and an absence of undercoordinated Ni or O. This is followed by the (104) facet with 1.66 J m^–2^. The (012) and (100) facets have similar surface energies with 1.88 J m^–2^ and 2.00 J m^–2^, respectively, while (110) is the highest energy surface in our calculations with 2.31 J m^–2^. In line with previous studies, the (104) facet is lower in energy than the (012) facet.^18,19^

**Figure 1:**
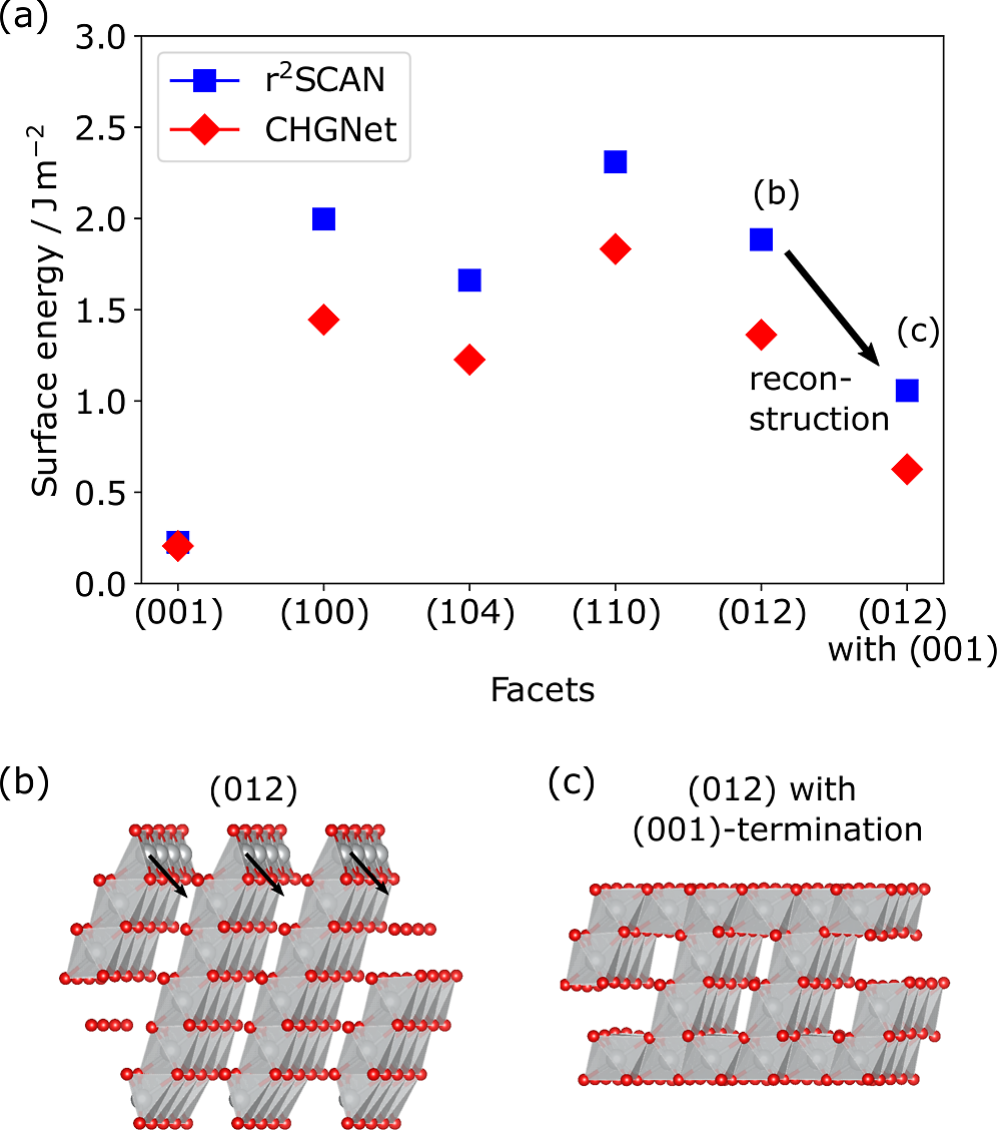
(a) Surface energies for different facets of NiO_2_ calculated using metaGGA DFT (blue) and the CHGNet MLIP fine-tuned to the same level of theory (red). The arrow indicates the reduction in surface energy for a (012) facet shown in (b) upon reconstruction to the (001)-like termination shown in (c) together with the evolution of molecular O_2_. Gray and red are Ni–O polyhedra and oxygen ions, respectively. Arrows in (b) indicate the movement of the surface Ni to sites in the Li-layer to form the structure presented in (c).

To test the accuracy of our fine-tuned MLIPs, which are used for MD simulations in the following sections, we also calculated the surface energies using these MLIPs. The ML model systemically underestimates the surface energy compared to DFT by 0.41 J m^–2^ (0.025 eV Å^–2^) on average but captures the relative ordering of surface energies correctly. This underestimation of surface energies by MLIPs is in line with previous reports of a systematic softening of the potential energy surface by MLIPs.^38,39^

The (012) surface has been observed experimentally^18,40,41^ and has been identified as a source of reduced cycling stability.^40,41^ To minimize the surface energy, the (012) facet can reconstruct with oxygen loss to a (001)-like termination via Ni migration into the empty lithium layers. This surface termination (Figure 1c) is rocksalt like, consistent with the experiment.^11^ Indeed, this reconstruction reduces the (012) surface energy to 1.06 J m^–2^ (1 atm, 300 K for the oxygen chemical potential calculation), which may act as a driving force for reconstruction separate from bulk thermodynamics. This densification process would include the loss of oxygen from the structure, also in line with experimental findings.^2,6^ We also computed a possible intermediate structure with half of the frontier Ni migrated into the octahedral sites, which we found to be unstable (see the Supporting Information). Interestingly, the structure shows some Ni migration above the Li layer, discussed below for our ML-MD simulations. Despite its higher energy, the structure could occur as a metastable intermediate state during a transformation process to the lowest-energy structure. While a thermodynamic driving force in the bulk material exists to form a metal-rich phase and molecular oxygen,^14^ our results suggest that there is also a strong thermodynamic driving force for the surface to reconstruct. While all relaxations have been carried out by fixing the simulation cell dimensions, we also performed MLIP relaxations allowing for selective relaxation of the (012) geometry along directions perpendicular to the surface normal. The expansion process yielded a reduction in total energy of −4.9 eV, which agrees with the proposed strain stabilization of reduced reconstructed phases proposed by Garcia et al.^42^ Our results highlight that mechanical strain is an important factor for the energetics of possible degradation pathways.

### 2.2 Kinetics of Phase Transformation

Previously, oxygen loss and molecular O_2_ evolution have been simulated on oxygen-overstoichiometric (012) surfaces.^20–22^ This coverage was predicted to be favorable under oxygen-rich conditions.^18^ However, we find that an oxygen-overstoichiometric NiO_2+δ_ surface with full oxygen coverage would not be favorable under experimentally achievable conditions for the delithiated material. We similarly do not expect an oxygen-rich termination in as-synthesized lithiated LiNiO_2_, which is discussed in the Supporting Information. Even if an oxygen-rich termination were present, we would expect the singly coordinated oxygen to be lost immediately, resulting in the thermodynamically favored oxygen-stoichiometric surface. Therefore, the loss of singly coordinated oxygen could be self-limiting and should not by itself explain the experimentally observed degradation.

We next performed ML-MD simulations on an oxygen-stoichiometric zero-dipole (012) surface with a half-monolayer oxygen termination at 800 K (Figure 2) to check for possible reconstruction. Within the first 50 ps, Ni ions move into the Li-layers before falling back to their original positions. At approximately 50 ps, a first drop in total energy slightly larger than 1 eV is observed, as all of the bottom frontier NiO_5_ pyramids flip into the lithium layers. Subsequently, the same happens to the top frontier NiO_5_ pyramids, which also leads to pronounced stabilization. The resulting structure is stable for the remaining simulation time scale (300 ps).

**Figure 2:**
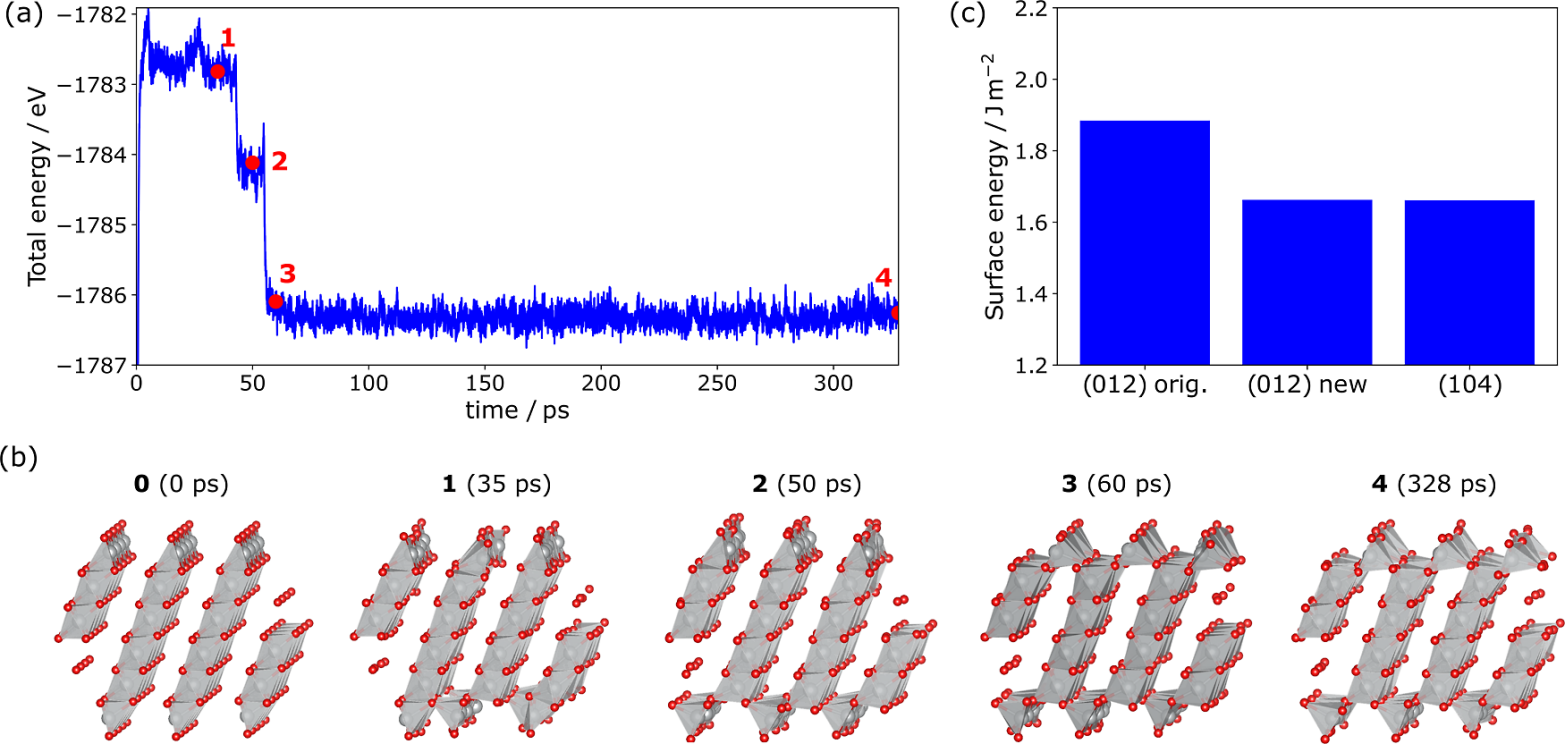
(a) Energy profile during the ML-MD simulation (*T* = 800 K) of the (012) facet of NiO_2_. (b) Structures highlighted in the energy profile in (a). (c) DFT-calculated surface energy of the original (012) facet, the new (012) facet, and the (104) facet for comparison.

To verify that this reconstruction is indeed energetically favorable compared to the pristine surface termination, we take the final sample structure from the MD trajectory and perform a geometry relaxation using a high level of theory (metaGGA) DFT. As shown in Figure 2c, this surface termination stabilizes the system by over 4 eV, which translates to a decrease in surface energy of 11.8%. With a surface energy of 1.66 J m^–2^, the reconstructed (012) surface is as stable as the (104) facet, which is also shown for reference. Bond-breaking as a simple metric to assess surface energies does not explain the significant decrease in surface energy for the reconstructed (012) facet since the nominal coordination of the surface Ni ions does not change. However, the slab energy also depends on their electronic structure and is influenced by the energy of the terminating polyhedra, which seem to gain an energetically more favorable geometric shape. This is reflected by the change in Ni–O bond lengths for both cases, as shown in Figure 3. Geometries of the surface NiO_5_ pyramids of both terminations are also shown. The long axial Ni–O bonds (denoted L1) are significantly longer in the original geometry (>2 Å). The equatorial short bonds toward the frontier oxygen (S1 in Figure 3) in the unreconstructed surface are shorter than on the reconstructed surface, indicating stronger bonding in the original surface.

**Figure 3:**
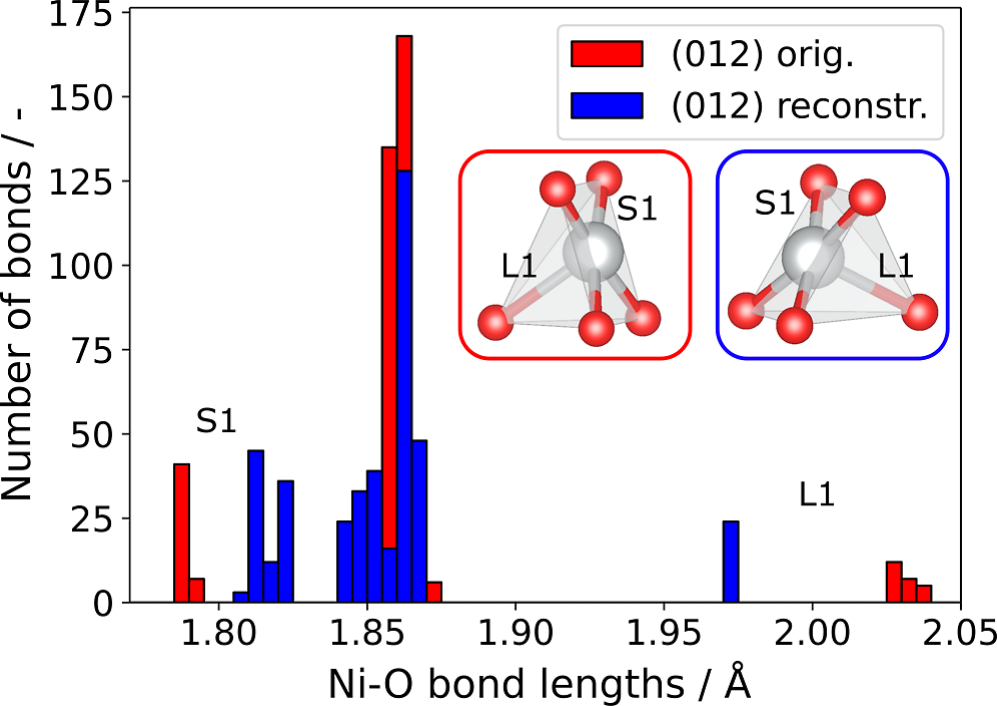
Histogram of Ni–O bond lengths occurring in the original (012) (red) and the new (012) reconstructed (blue) facet. The short (S1) and long (L1) bonds can be attributed to the top and bottom Ni-pyramids indicated in the inset figures for the original structure (left) and the reconstructed structure (right). The new reconstruction shows shorter axial bonds L1 and longer equatorial bonds S1.

The newly obtained surface termination was very stable in subsequent simulations, showing no further reconstructions even at higher temperatures. This pathway is in line with the geometry relaxation of the intermediate transition structure discussed in Section 2.1 and presented in the Supporting Information, which showed signs of this transformation even in static relaxation calculations. The ML-MD trajectory samples the complete reconstruction. While the reconstruction and degradation pathways of the (012) surface will depend on its oxygen termination and off-stoichiometry, we study the oxygen-stoichiometric half-monolayer configuration. This is the lowest-energy termination in oxygen environments spanning from synthesis through operation, as much as the chemical potential of oxygen can be defined in a battery by possible oxygen acceptors.

Similar to the (001)-like surface termination discussed in Section 2.1, we also relaxed the new reconstructed (012) surface structure using our MLIP model without constraints in the directions perpendicular to the surface normal, i.e., allowing for strain relative to bulk NiO_2_, but found only a slight stabilization (−0.129 eV total energy). Therefore, mechanical strain is less important for this specific pathway.

To further check the validity of the ML-predictions, Figure 4 shows the evolution of magnetic moments of Ni and oxygen during the ML-MD simulations and its comparison to the magnetic moments obtained through a DFT relaxation of the final reconstructed structure (Figure 4a). We expect magnetic moments near 1.6 μ_B_ (spin *S* = 1), 0.86 μ_B_ (*S* = 1/2), and approximately 0 μ_B_ (*S* = 0) for formally +2 Ni, +3 Ni, and +4 Ni, respectively.^31^ The majority of Ni is around 0 μ_B_ (Figure 4c), thus consistent with the formal +4 oxidation state. Furthermore, a fraction of magnetic moments between 0.8 μ_B_ and 1 μ_B_ is observed, which indicates +3 Ni. Most oxygen ions have an expected magnetic moment around 0 μ_B_, while further peaks around 0.2 μ_B_ and 0.5 μ_B_ are observed (Figure 4d). The ML-MD magnetic moments are in good agreement with those obtained from DFT (Figure 4e,f): the Ni ions can be found mainly in formal +4 and +3 oxidation states. Most oxygen ions have a formal 2− oxidation state (near-zero magnetic moments), with some having a higher magnetic moment of 0.5 μ_B_.

**Figure 4:**
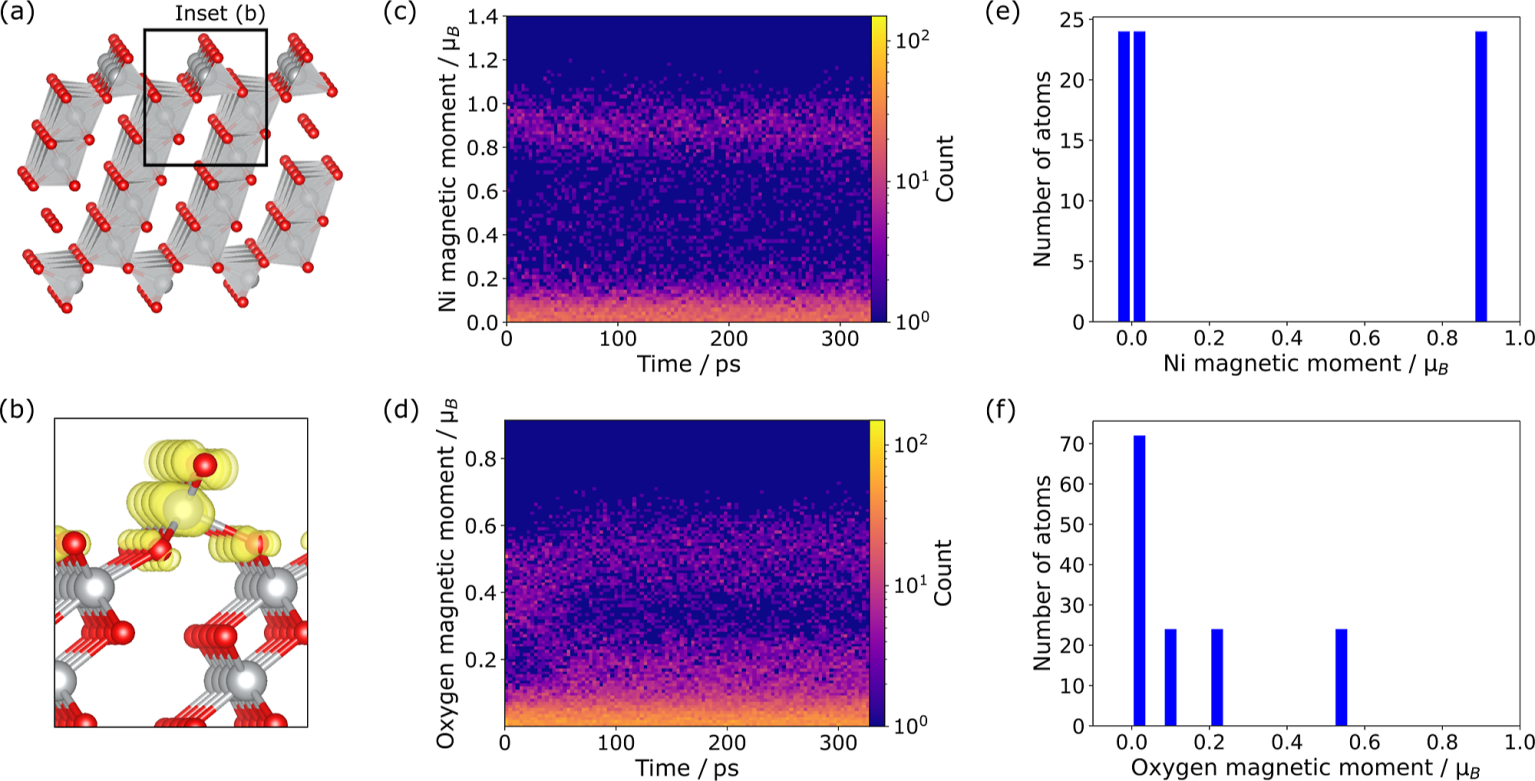
(a) Reconstructed (012) surface slab of NiO_2_ after geometry relaxation with Ni in gray and O in red. (b) Spin-density plot at the top of the structure with yellow denoting a positive spin density. (c) Histogram of Ni magnetic moments during ML-MD run from CHGNet. Colors indicate the number of occurrences of the magnetic moments, i.e., lighter color represents more atoms with this magnetic moment. (d) Oxygen magnetic moments during ML-MD run from CHGNet. (e) Number of occurrences of Ni magnetic moments in final structure from DFT calculation. (f) Number of occurrences of oxygen magnetic moments in the final structure from DFT calculation. The number of occurrences sum up to the total count of Ni and O atoms in (e) and (f), respectively.

Overall, most ions are in the formal oxidation states expected for NiO_2_. As shown in Figure 4b, the higher spin densities concentrated on the surface atoms for both Ni and oxygen. During the reconstruction, the distribution of oxygen magnetic moments changes (Figure 4d) at around 50 ps, i.e., when the stable surface reconstruction first forms (Figure 2a). This suggests that surface reconstruction modifies the state of surface oxygen. The higher oxygen magnetic moments indicate oxidation of the under-coordinated oxygen at the surface. We discuss the consequences for surface stability below.

The perturbation to oxidation states at the surface is also seen in the projected density of states (DOS) in Figure 5a. In the total DOS, the Ni 3d and the O 2p orbitals overlap and contribute to states just below the Fermi level. The Ni ions in the bulk have no unpaired electrons, which is clearly visible from its symmetric band structure (blue in Figure 5b). The surface Ni (red in Figure 5b) shows a contribution from unoccupied spin-down states due to the presence of unpaired electrons. Together with the MD results, this analysis of the surface electronic structure suggests that the formal oxidation of oxygen is largely associated with its (under)­coordination at surfaces rather than with the nature of bulk Ni–O bonding.

**Figure 5:**
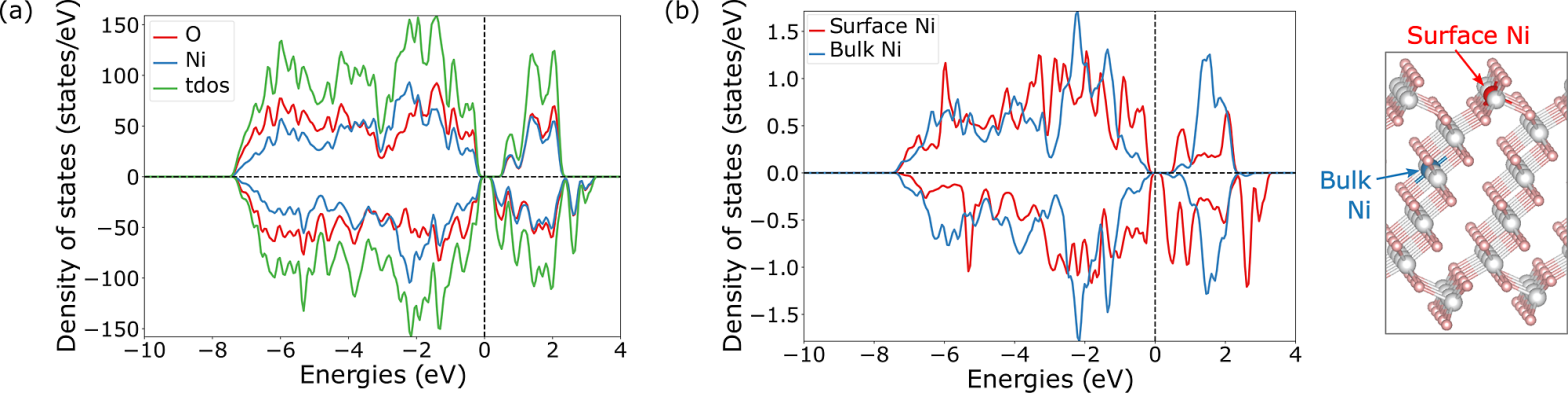
Projected DOS of the reconstructed (012) termination of NiO_2_ for (a) Ni and O and (b) for Ni disaggregated between the surface (under-coordinated, red line) and the bulk (blue line). The corresponding Ni in the structure are highlighted in red (surface) and blue (bulk). Fermi energy is shifted to 0 eV.

### 2.3 Energetics of Oxygen Vacancy Formation

An important defect that might serve as an intermediate in surface degradation is the oxygen vacancy, V_O_. Therefore, we next investigate the formation energy of oxygen vacancies in the pristine and newly found surface termination.

Figure 6a shows the oxygen vacancy formation energies in layered NiO_2_ at standard conditions (300 K and 1 atm) in the bulk structure and at three surfaces: the (104) facet, the standard (012) facet, and the newly formed (012) surface termination. The oxygen vacancy formation energy in the bulk is much higher than on any surface, which is consistent with previous reports^43,44^ and the intuition that the defect formation energy scales with the number of broken bonds: three Ni–O bonds are broken per V_O_ in the bulk versus at most two bonds on the oxygen-stoichiometric surfaces examined here. For comparison, the formation energy of a single oxygen vacancy on the (104) surface is 0.09 eV higher than that on (012). This result is consistent with the higher stability and lower surface energy of the (104) surface and is line with previous calculations^18^ and experiments.^41^ Oxygen vacancies also form more easily on the newly found (012) termination with a formation energy of 0.19 eV, than on the unreconstructed neutral (012) surface. The oxygen vacancy formation energy decreases by nearly 0.5 eV on the newly found surface termination versus the unreconstructed (012) surface, despite identical formal numbers of bonds broken. This can be rationalized by the longer, and thus weaker, Ni–O bonds in the top Ni–O polyhedra of the new termination shown in Figure 3. This is consistent with the additional oxidation of surface oxygen during this reconstruction as evidenced by the rise of surface oxygen magnetic moments toward 0.5 μ_B_ during reconstruction (Figure 4d).

**Figure 6:**
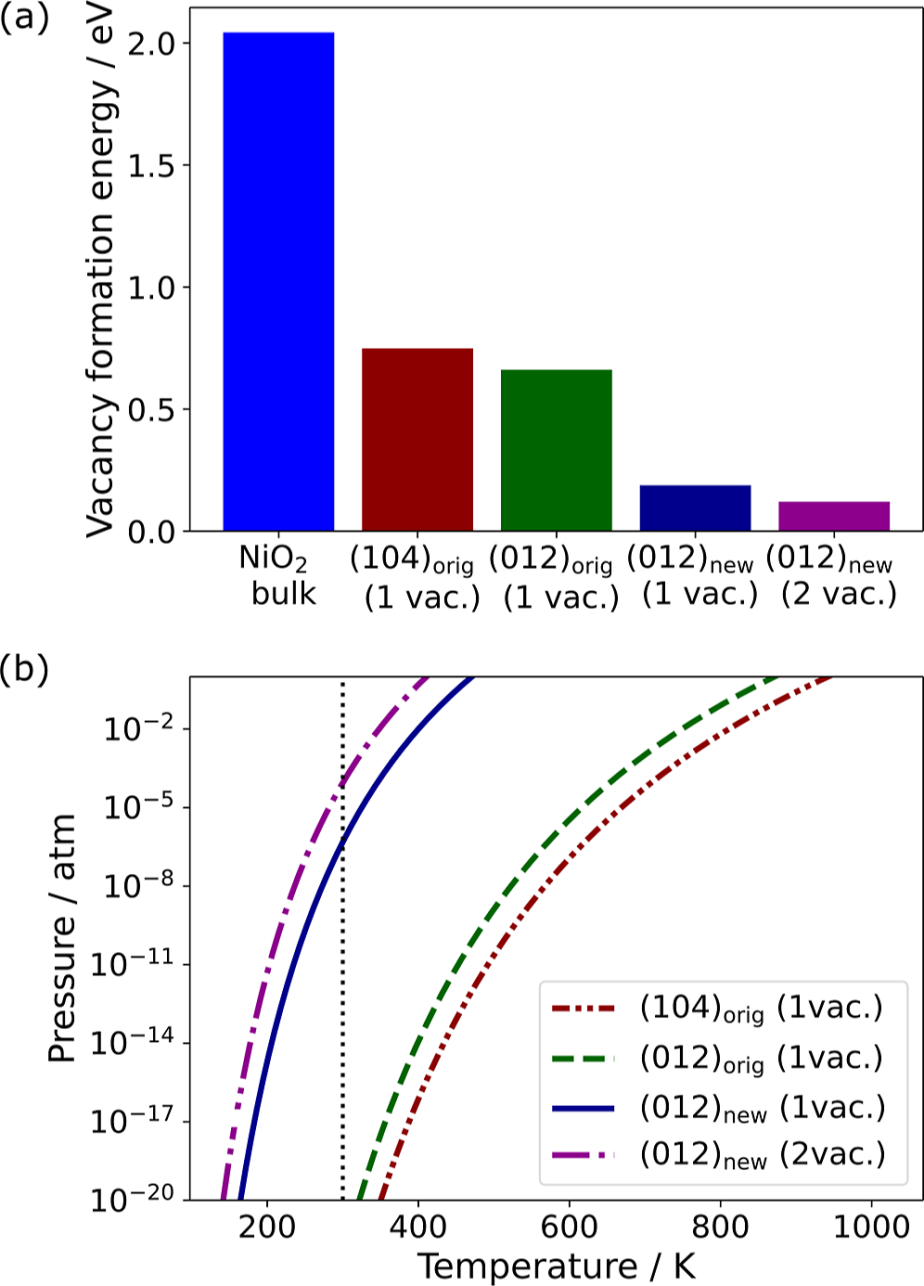
(a) Oxygen vacancy formation energies in the bulk of NiO_2_, the pristine (104) and (012) surfaces, and the new (012) termination at 300 K and 1 atm. (b) Stability region of the surface vacancy defects shown in (a) as a function of temperature and pressure. Oxygen vacancy formation is favorable in regions to the right side of the respective lines in (b). The vertical dotted line in (b) indicates 300 K.

The formation of two adjacent oxygen vacancies has a lower formation energy of 0.12 eV per vacancy. This suggests that oxygen loss via vacancy formation could be autocatalytic; i.e., since the formation energy is lowered by the presence of a neighboring vacancy, an initial vacancy could catalyze the formation of further vacancies.

Figure 6b shows the regions of stability in temperature and the effective oxygen partial pressure (*p*_O_2__) of oxygen vacancies on the relevant surfaces. On the new (012) termination, the formation energy becomes negative, and hence, V_O_ formation would become favorable, at effective oxygen partial pressures *p*_O_2__ below 4.9 × 10^–7^ atm at 300 K, or above 471 K at 1 atm *p*_O_2__. This closely matches the temperatures at which charged Ni-rich oxide cathodes decompose thermally, typically about 460–470 K from thermogravimetric analysis experiments.^4,13^ Our simulations suggest that a surface-initiated self-propagating O_2_ evolution is a plausible mechanism for the decomposition of the charged cathode material.

Furthermore, although the V_O_ formation energy refers to a 1 atm *p*_O_2__ standard state, the effective *p*_O_2__ in the closed system in contact with the electrolyte could be lower during cell operation (see for example the discussion in the Supporting Information of ref 45). The effective *p*_O_2__, set by oxygen acceptors in solution, may be sufficiently low to render vacancy formation favorable at room temperature.

The oxygen vacancy formation energies calculated here are higher than values commonly reported for charged LiNiO_2_ or Ni-rich NMC.^18,43,44,46^ The choice of DFT functional and application of a van der Waals correction can have a significant impact on the calculated energy,^44^ and most previously reported values have been calculated at the GGA + *U* level of theory. To verify whether the difference is attributable to the level of theory, we also calculated the V_O_ formation energy in bulk NiO_2_ and on the oxygen-stoichiometric (012) surface using the PBE + *U* functional (*U*_eff_ = 5.96 eV^23,42^ ). Indeed, our PBE + *U* results show much lower defect formation energies (Supporting Information). The metaGGA exchange correlation functionals such as SCAN and r^2^SCAN have been shown to represent metal–oxygen bonding more accurately and without the need of the hyper-parameter +*U* owing to their nonempirical nature.^23,27,47,48^ Due to the previously discussed agreement with experimental observations and the absence of such a hyper-parameter, we consider our vacancy calculations physically plausible.

Quantitative benchmarking of our first-principles results to experimentally obtained oxygen gas evolution rates is tenuous, as they result from the convolution of many factors. These include an intrinsic surface reaction rate dependent on solvent composition, active surface area (potentially influenced by a carbon-binder phase), or grain-boundary-mediated and defect-mediated O_2_ evolution. The calculated vacancy formation energies are, nevertheless, in qualitative agreement with previous experimental findings,^2^ which conclude that increasing nickel content in the Li­(Ni,Mn,Co)­O_2_ series delays the onset of O_2_ evolution to lower remaining lithium fractions in the presence of solvent. This is consistent with the small but positive oxygen vacancy formation energies that we compute in the absence of solvent.

As all of our r^2^SCAN simulations yield positive vacancy formation energies, the question of how the presence of an electrolyte might alter the effective chemical potential to create a vacancy is therefore very important. Vacancy formation could plausibly occur as a result of interaction with the electrolyte^49,50^ at finite pressures and temperatures, which is not captured by our current approach and warrants future investigation. Specifically, molecular oxygen acceptors in the electrolyte could set an effective chemical potential of oxygen during battery operation and, in this way, affect the surface oxygen off-stoichiometry. However, we reiterate the necessity to use a level of theory that accurately captures the underlying physics to extend the study of surface chemistry to solvent reactivity.

While we do not study the interface with the electrolyte, we investigate how an oxygen vacancy might affect the subsequent degradation process and structural stability. Therefore, we perform ML-MD simulations at an elevated temperature (900 K) on dipole-free slabs containing two symmetric vacancies and compare the (104) surface and the reconstructed (012) surface.

In both cases, Ni ions migrate to the empty alkali layer (Figure 7a,b). The trajectories demonstrate that the presence of frontier oxygen vacancies leads to subsequent migration of Ni ions from their initial positions into the alkali-layer. On the (012) surface, the Ni ion migrates to an octahedral site in the alkali layer, whereas at the (104) surface, the frontier Ni atoms move to tetrahedral sites. This occurs already during initial relaxation with further Ni migration to tetrahedral sites throughout the MD simulation. By relaxing structures sampled in MD with DFT, we verified that these transition-metal migration events lead to energetically more favorable structures. Since these Ni migration events were sampled through limited MD runs, the real rates of these processes cannot be extremely slow, and they can represent kinetically plausible initial steps of degradation of delithiated LiNiO_2_.

**Figure 7:**
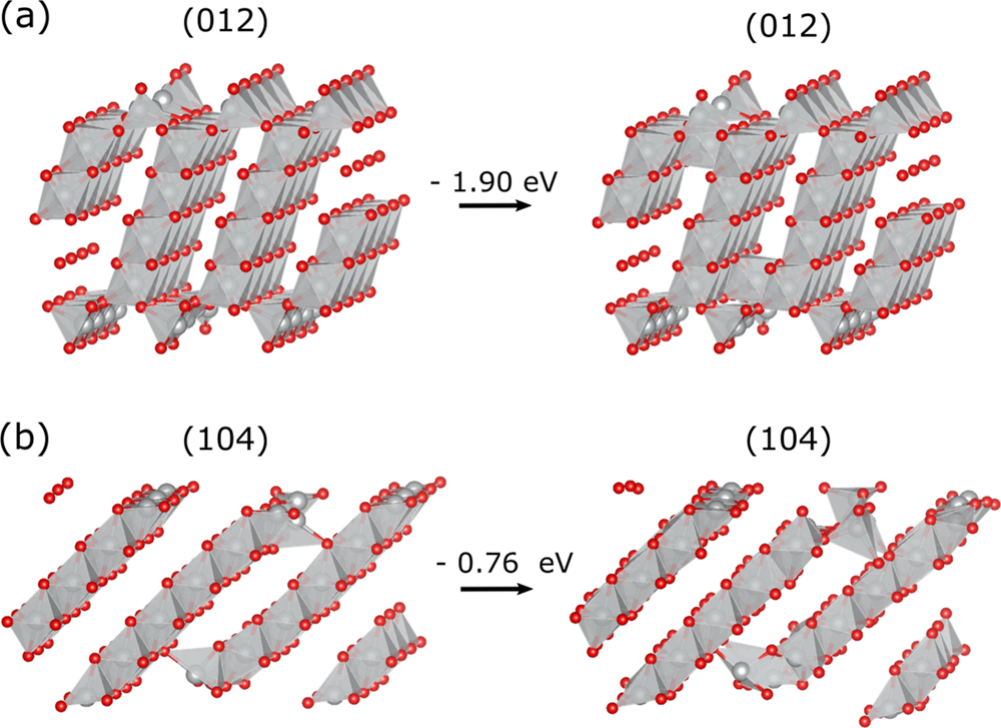
(a) (012) facet with two symmetric vacancies on each side. Ni-migration into octahedral sites sampled with ML-MD lowers resulting energy by 1.9 eV when relaxed with DFT. (b) Ni migration in the (104) facet into tetrahedral sites lowers the total energy by 0.8 eV when relaxed with DFT.

Our results suggest that following the formation of V_O_, further degradation steps are immediately energetically favorable and could propagate toward thermodynamically stable products, e.g., spinel or rocksalt phases. Extending previous studies on oxygen vacancy formation in layered oxide cathode materials,^15,18^ our study proposes a novel transformation pathway from a layered crystal structure toward a rocksalt-like surface structure that is mediated by the presence of oxygen vacancies. We demonstrate the thermodynamic favorability of an intermediate (012) termination, on which oxygen evolution is facilitated, ultimately leading to subsequent Ni migration. Although beyond the scope of the current study, accounting for electrolyte solvents and salts will further assist in the study of possible degradation mechanisms and will be the subject of future investigation. Nevertheless, our approach is more easily tractable and mimics fast thermal stability tests employed experimentally for the same reason.^4,8,13^ Indeed, as with thermal stability measurements, our work already reveals both a thermodynamic driving force for surface reconstruction that is complementary to bulk energetics and a kinetically feasible pathway of Ni migration.

## 3 Conclusion

The structural degradation pathways of NiO_2_ cathode surfaces at the top of charge are investigated using machine-learning molecular dynamics (ML-MD) combined with metaGGA DFT. We find a surface-based driving force to reconstruct rocksalt-like facets concomitant with oxygen loss. The simulations indicate a novel reconstruction of the oxygen-stoichiometric (012) surface via flipping of frontier NiO_5_ pyramids into the alkali metal layer. Furthermore, oxygen vacancy formation on the reconstructed (012) termination is more favorable than on the original facet. The lower vacancy formation energy is due to longer equatorial Ni–O bonding of the surface Ni and oxygen ions and higher oxidation of the under-coordinated oxygen. Notably, our calculated vacancy formation energies closely correspond to temperatures at which charged cathodes decompose thermally, suggesting that self-sustaining oxygen evolution initiated at the surface is a plausible mechanism for the decomposition. The results show that the presence of oxygen vacancies can lead to kinetically feasible Ni migration and therefore may be a first step in a degradation pathway toward thermodynamically stable products. Our study can serve as a building block toward rigorous simulations of solvent interactions with the surfaces of charged cathodes at relevant levels of first-principles theory and starting with thermodynamically correct stoichiometries.

From a practical perspective, oxygen evolution needs to be addressed, as it leads to further transformation, which can be mitigated by coatings or doping of the material. Our study informs a design principle for such coatings: to passivate any under-coordinated oxygens, either at surfaces as simulated here or at grain and domain boundaries. Additionally, the crystal structure should be optimized during synthesis toward more stable surface terminations and to mitigate such internal boundaries.

## 4 Methods

Spin-polarized ab initio calculations have been performed using the Vienna Ab Initio Simulation Package (VASP) using a plane-wave basis set and projector-augmented-wave formalism. We used the metaGGA functional r^2^SCAN^29^ with the revised Vydrov-van Voorhis (rVV10 with *b* = 11.95 and *c* = 0.0093) nonlocal dispersion correction^51^ as in our work focusing on bulk LiNiO_2_.^31^ We use an energy cutoff of 700 eV with an electronic convergence criterion of 10^–5^ eV using the “normal” precision setting for the reciprocal-space mesh resolution and a Gaussian smearing width of 0.05 eV. A Γ-centered *k*-point mesh with a spacing of 0.25 Å^–1^ was used. For ionic relaxation, a force cutoff of 10^–2^ eV Å^–1^ was used. If not stated otherwise, all geometry relaxations were performed by doing an electronic optimization with up to 250 iterations and subsequent ionic relaxation for better convergence. For comparison, some calculations have been performed using the PBE functional with a Hubbard *U* correction. We apply Dudarev’s method with an effective *U*-value of 5.96 eV.^23,42^ Molecular orbital projection was done using LOBSTER^52^ using the pbeVaspFit2015 basis set. Calculations have been performed on the ARCHER2 UK National Supercomputing Service.^53^

ML molecular dynamics simulations have been carried out using Crystal Hamiltonian Graph Neural Network (CHGNet) as the interatomic potential.^33^ We fine-tuned the model on 9298 structures sampled from bulk and surface relaxations from states of lithiation from LiNiO_2_ to NiO_2_. We fine-tuned for 154 epochs using an MSE loss criterion with weights of 1, 100, 1, and 1 for energies, forces, stresses, and magnetic moments, respectively. The batch size was set to 2, and we used the Adam Optimizer with an initial learning rate of 10^–3^. ML-MD was carried out on the University of Oxford Advanced Research Computing (ARC) facility^54^ using an *NVT* ensemble. Structure relaxations were performed using the BFGS algorithm and a force cutoff of 10^–2^ eV Å^–1^.

The surface energies have been calculated according to

1 $$\gamma =\frac{1}{2A}\left[G_{s}-\frac{n_{Ni}^{s}}{n_{Ni}^{b}}G_{b}-\Gamma_{O}\mu_{O}\left(T,p\right)\right]$$

with the cross-sectional area *A*, the total energies of the surface slab *G*_s_ and the bulk *G*_b_, as well as the number of Ni *n*_Ni_^s^ and *n*_Ni_^b^ in the slab and in the bulk, respectively. The surface energy also depends on the oxygen chemical potential μ_O_ in excess or shortage of oxygen ions Γ_O_ with respect to the stoichiometric surface slab composition^55^

2 $$\Gamma_{O}=n_{O}^{s}-\frac{n_{Ni}^{s}}{n_{Ni}^{b}}n_{O}^{b}$$

with *n*_O_ as the number of oxygen and *n*_Ni_ as the number of Ni in the slab and the bulk, which are denoted by the superscripts “s” and “b”. Note that if the slab is stoichiometric, Γ_O_ equals zero, and the surface energy does not depend on the oxygen environment. The oxygen chemical potential is calculated as

3 $$\mu_{O}\left(T,p\right)=\frac{1}{2}E_{O_{2}}+\Delta \mu_{O}$$

with *E*_O_2__ as the DFT ground state energy of an oxygen molecule. Δμ_O_ is calculated as in

4 $$\Delta \mu_{O}=\frac{1}{2}\left(H_{0}+dH-T\left(S_{0}+dS\right)+RT\;\ln\left(\frac{p}{p_{0}}\right)\right)$$

with d*H* = *C*_*p*_(*T* – *T*_0_), d $S=C_{p}\;\ln\left(\frac{T}{T_{0}}\right)$ , *T*_0_ = 298 K, *p*_0_ = 1 atm, *H*_0_ = 8700 J mol^–1^, *S*_0_ = 205 J (mol K)^−1^, and *C*_*p*_ = 3.5·*R*, where *R* is the universal gas constant.^17,19^ The oxygen vacancy formation energy per charge-neutral vacancy defect was calculated according to eq 5.

5 $$dE=\frac{1}{n_{vac}}\left(E_{{NiO}_{2-\delta }}-E_{{NiO}_{2}}+n_{vac}\mu_{o}\right)$$

We calculate the defect formation energy per vacancy considering the DFT energies of a defect-free and the defect structure as well the ground-state energy and chemical potential of oxygen. Defects were created symmetrically on the slabs to obtain a dipole-free geometry for geometry optimization; therefore, we need to consider the total number of vacancies *n*_vac_.

Data analysis and preparation have been carried out using the python libraries ASE (atomistic simulation environment)^56^ and Pymatgen.^57^ VESTA^58^ and Vaspkit^59^ were used for structure visualization and generating spin-density plots.

## Supplementary Material



## Notes

PBE + *U* oxygen vacancy formation energies; MLIP evaluation; convergence checks for surface energies and defect formation energies; calculation of intermediate structure in transition pathway; CHGNet relaxations of surfaces in directions perpendicular to the surface normal; and total energies and structures for oxygen vacancy defects in different sites (PDF)
