# Supplementary material for: Probing Surface Degradation Pathways of Charged Nickel-Oxide Cathode Materials Using Machine-Learning Interatomic Potentials
Source: ACS Appl Mater Interfaces. 2025 Sep 25;17(40):56612–20. doi: 10.1021/acsami.5c11818 (PMC12516693; doi:10.1021/acsami.5c11818)
Supplement: Supplementary file 1 [file am5c11818_si_001.pdf]

Supporting Information: Probing surface degradation pathways of charged nickel-oxide cathode materials using machine-learning interatomic potentials

Svenja Both[1,2,3,*], Andrey D. Poletayev[3,4,*], Timo Danner[1,2], Arnulf Latz[1,2,5], and M. Saiful Islam[3,4,*]

[1]German Aerospace Center, Institute of Engineering Thermodynamics, 89081 Ulm, Germany
[2] Helmholtz-Institute Ulm for Electrochemical Energy Storage, 89081 Ulm, Germany
[3] Department of Materials, University of Oxford, Oxford, OX1 3PH, United Kingdom
[4]The Faraday Institution, Harwell Science and Innovation Campus, Didcot, OX11 0RA, United Kingdom
[5]Institute of Electrochemistry, Ulm University, 89081 Ulm, Germany
[*]E-mail: svenja.both@dlr.de, andrey.poletayev@gmail.com, saiful.islam@materials.ox.ac.uk

1 Comparison of r^2SCAN and PBE+U functionals

For comparison, we performed PBE+U (U_{eff}=5.96 eV) calculations to obtain vacancy formation energies in the bulk and on the (012)-surface. Fig. S1 shows the resulting vacancy formation energy. This calculation results in a vacancy formation energy of +0.96 eV in the bulk ($Ni_{36}O_{72}$) and -0.06 eV on the (012) surface slab. The r^2SCAN values reported in the main manuscript are +2.04 eV and +0.66 eV for the bulk and the (012) facet, respectively. Both sets of energies include the chemical potential of oxygen as calculated in the main manuscript at 300 K and 1 atm p_{O_2}. These values are significantly smaller than the ones obtained using r^2SCAN. PBE+U underestimates oxide binding energies [1–4] and often, an empirical correction of -1.36 eV per O_2 molecule is applied when using this functional [5–7]. In addition, the electronic structure depends on the applied +U value, which varies by more than 1 eV during a full (de-)lithiation [8].

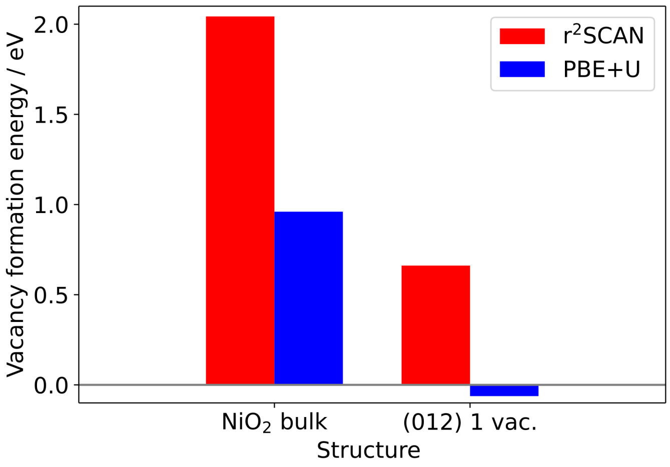

Figure S1: PBE+U oxygen vacancy formation energy for NiO_2 for bulk and surface calculations at 300 K and 1 atm.

2 Dependence of surface energy on oxygen chemical potential

As discussed in the main manuscript, we use a stoichiometric and dipole-neutral slab model for the (012)-surface with a half-monolayer termination of oxygen. The surface energy as a function of chemical potential can be calculated according to equations (1)-(4), see Methods in the main manuscript.

Delithiated state. We first calculate the dependence of surface energy for different oxygen coverages of the delithiated (012) surface. We observe that for oxygen chemical potentials μ_O above approximately -1 eV, the stoichiometric (012) surface slab would be the most favorable. In very oxygen-poor environments, the 1/4 ML coverage would be favorable. However, even in oxygen-rich conditions, our simulations demonstrate how a non-stoichiometric, fully-covered surface would not be favored.

Lithiated state. To mimic conditions during synthesis, we perform this calculation on a fully lithiated (012) surface as well. We find that the stoichiometric (1/2 ML) surface would be stable in most conditions as well. The 1ML termination, frequently reported as a starting point for MD studies, would only be favorable at very oxygen-rich conditions, which is line with literature [9]. However, at 700 °C and 1 atm the oxygen chemical potential would be -1.06 eV, which is far below the stability region of this surface termination. Also note that Li moved out of the slab during relaxation for the 1ML configuration, which might lower its energy.

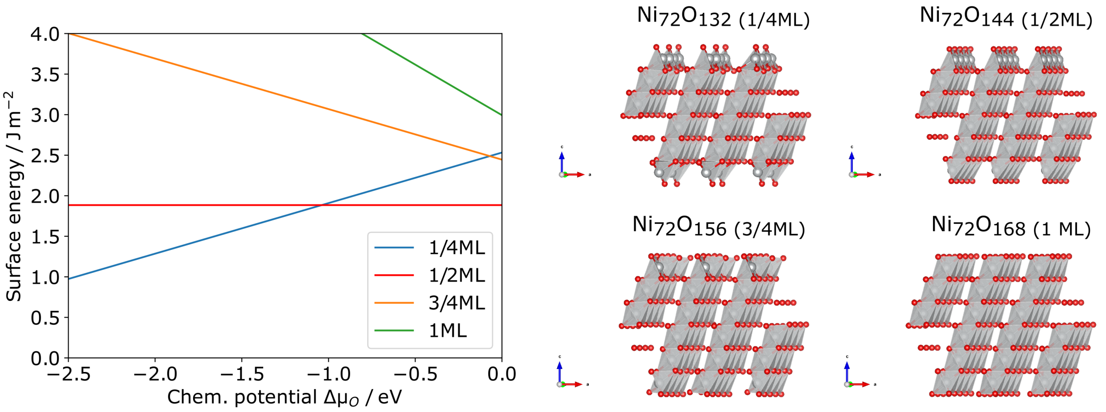

Figure S2: Surface energy of the delithiated (012) facet of NiO_2 for different coverages of oxygen.

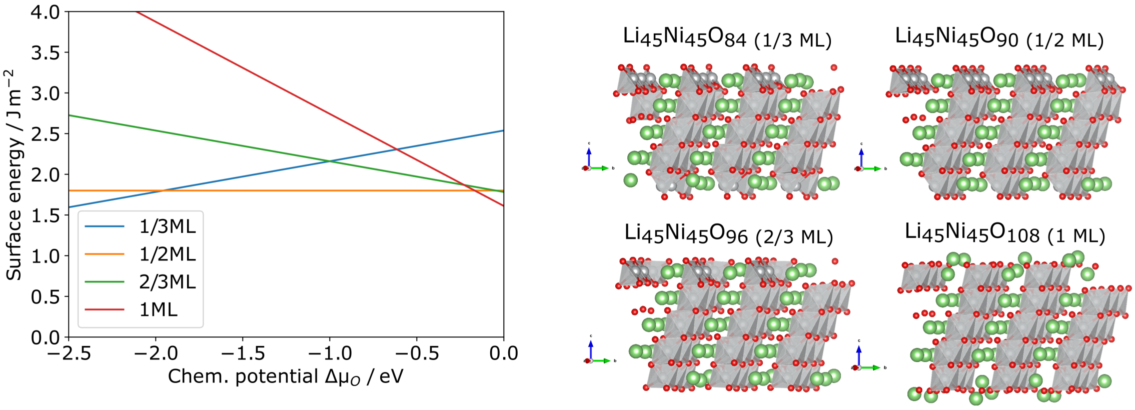

Figure S3: Surface energy of the lithiated (012) facet of $LiNiO_2$ for different coverages of oxygen.

3 Intermediate structure

As mentioned in the main manuscript, we calculated the (012) facet without any reconstruction as well as a (001) like surface termination from it, which was shown to be energetically favorable. In an attempt to understand this pathway better, we also calculated one possible intermediate structure (middle structure) with half of the Ni-layer moved into octahedral sites of the Li-layer (still stoichiometric composition). Even though we consider this pathway non-favorable due to its high energy (-1806 eV compared to original (012)-termination with -1825.52 eV), the relaxed structure shows Ni-movement in line with our CHGNet-predictions of a surface reconstructed layer. Furthermore, even though the total energy is higher, it could occur as a meta-stable intermediate structure during a transformation process.

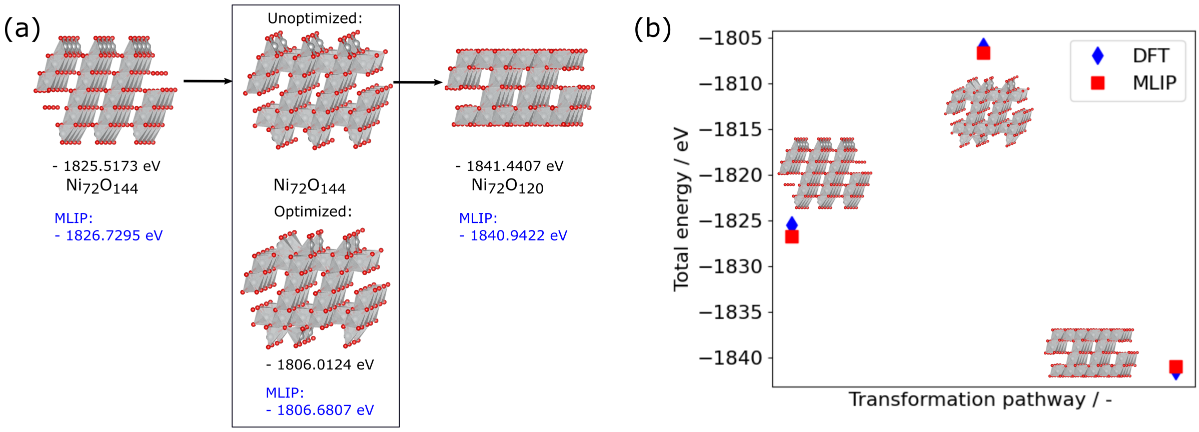

Figure S4: (a) Possible intermediate structure in degradation pathway with total energies obtained from DFT and MLIP calculations. The non-stoichiometric surface includes the oxygen chemical potential at 300 K and 1 atm for the oxygen ions in shortage. (b) Graphical representation of the total energies and structures shown in (a).

4 Relaxation in lattice directions perpendicular to surface normal

Table S1 summarizes the total energies as well as lattice parameters after a relaxation of the slabs in both a and b directions, i.e., perpendicular to the surface normal. Values in brackets refer to the total energy and lattice constants after a geometry optimization of the initial structure with fixed cell dimensions. The initial structure is the relaxed structure as obtained from DFT. Note that these calculations have been performed using CHGNet.

Structure	Surface	Total Energy / eV	a / Å	b / Å	Engine
$Ni_{72}O_{144}$	(012) original	-1826.8953	14.13952	10.98814	CHGNet
		(-1826.7295)	(14.15468)	(11.10787)	CHGNet
$Ni_{72}O_{144}$	(012) new	-1830.5708	14.13170	11.02545	CHGNet
		(-1830.4417)	(14.15468)	(11.10787)	CHGNet
$Ni_{72}O_{120}$	(012) with (001)	-1702.7360	14.96385	11.26397	CHGNet
		(-1697.8326)	(14.15468)	(11.10787)	CHGNet

Table S1: Total energies of surface slabs for relaxation in both a and b direction as well as for structure relaxation of the initial structure with fixed cell dimensions (values in brackets). All calculations have been carried out using CHGNet.

5 Comparison of different vacancy sites

We compare different vacancy positions for the new surface reconstruction. From the different possible vacancy sites, we chose the one that is lowest in total energy for our analysis. As shown in Fig. S5, a vacancy on the frontier, two-coordinated oxygen of the slab is more stable than on sites "B" and "C", which are coordinated by three transition metals. This is plausible since vacancy formation in site "A" only requires breaking of two bonds to neighboring Ni, while both sites "B" and "C" would require 3 bonds to break.

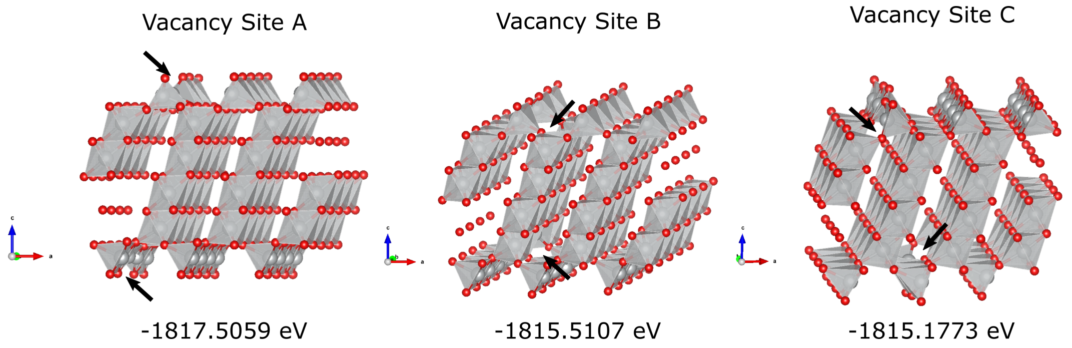

Figure S5: Total energies of structures with oxygen vacancies in different sites.

6 Evaluation of the MLIP

The MLIP was evaluated on the training data set in terms of energy per atom and forces in x, y and z direction. The mean average error (MAE) of the energy per atom is 10 meV/atom and the MAE of forces in x and y direction are 10 eV/Å and in z direction 12 eV/Å.

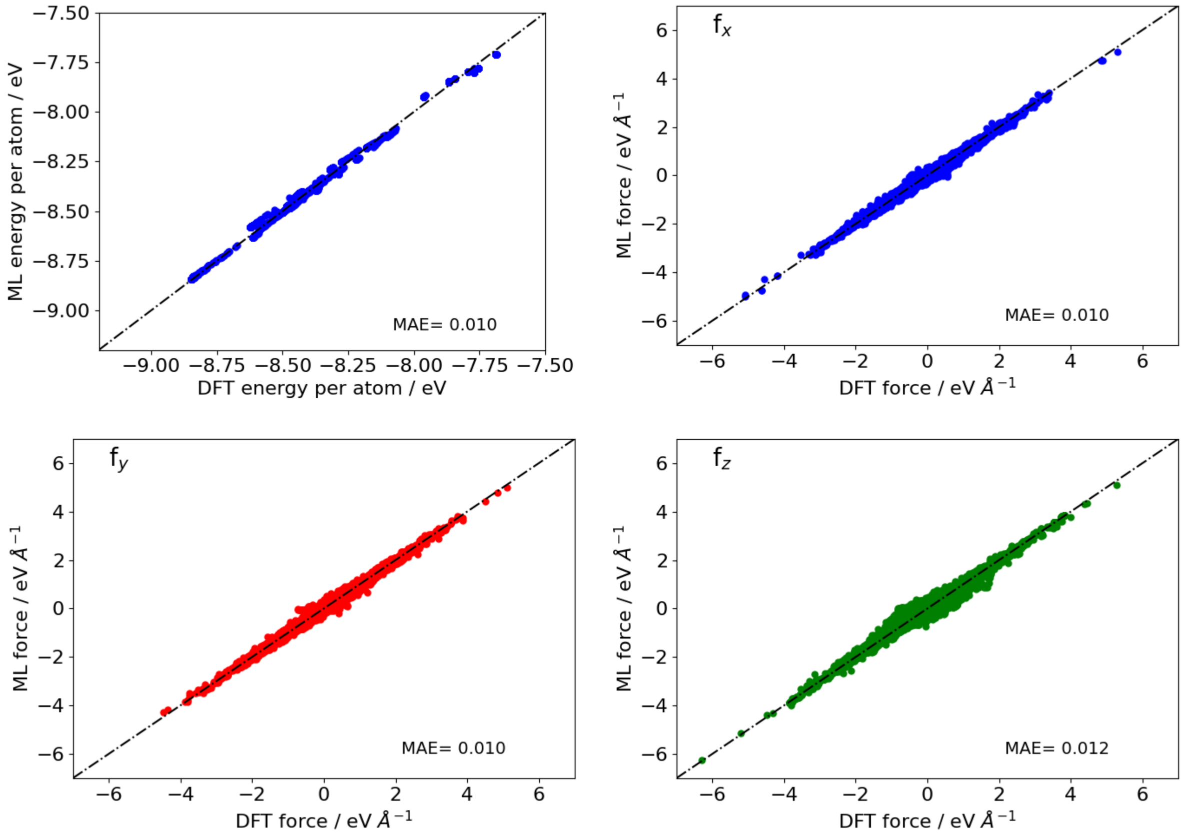

Figure S6: Comparison of energy and force components predicted by DFT and the MLIP with indicated mean absolute error (MAE).

7 Convergence checks

To check the convergence of our calculations, we compute the surface energies and defect energies of the standard (012) facet using different k-point meshes and vacuum thicknesses. The calculations in main manuscript have been performed using 20 Å of vacuum (10 Å on each side of the slab) and a k-point spacing of 0.25 $Å^{-}1$.

7.1 Surface energy

Impact of vacuum size

- Surface energy with vacuum of 20 Å (main manuscript): 1.8845 J/m^2
- Surface energy with vacuum of 30 Å: 1.8820 J/m^2
- Surface energy with vacuum of 40 Å: 1.8813 J/m^2

Impact of k-point mesh

- K-point spacing of 0.25 $Å^{-}1$ results in 2x3x1 mesh (main manuscript): 1.8845 J/m^2
- K-point mesh 3x3x1: 1.8843 J/m^2

7.2 Vacancy formation energy

- Vacancy formation energy in main manuscript: 0.661 eV
- Vacancy formation energy with 30 Å vacuum: 0.684 eV
- Vacancy formation energy with 3x3x1 k-point mesh : 0.662 eV

In summary, the applied k-point spacing and vacuum thickness lead to results within sufficient accuracy for both surface energy and vacancy formation energy.

References

(1) L. Wang, T. Maxisch and G. Ceder, *Phys. Rev. B*, 2006, **73**, 195107.

(2) J. Vinckeviciute, D. A. Kitchaev and A. Van Der Ven, *Chemistry of Materials*, 2021, **33**, 1625–1636.

(3) D. A. Kitchaev, J. Vinckeviciute and A. Van der Ven, *Journal of the American Chemical Society*, 2021, **143**, 1908–1916.

(4) Y. Zhang, D. A. Kitchaev, J. Yang, T. Chen, S. T. Dacek, R. A. Sarmiento-Pérez, M. A. Marques, H. Peng, G. Ceder, J. P. Perdew and J. Sun, *npj Computational Materials*, 2018, **4**, 9.

(5) K. Min, S.-W. Seo, Y. Y. Song, H. S. Lee and E. Cho, *Physical Chemistry Chemical Physics*, 2017, **19**, 1762–1769.

(6) X. Li, Q. Wang, H. Guo, N. Artrith and A. Urban, *ACS Applied Energy Materials*, 2022, **5**, 5730–5741.

(7) J. Cheng, L. Mu, C. Wang, Z. Yang, H. L. Xin, F. Lin and K. A. Persson, *Journal of Materials Chemistry A*, 2020, **8**, 23293–23303.

(8) P. M. Kowalski, T. Bornhake, O. Cheong, N. Dohrmann, A. L. Koch Liston, S. K. Potts, A. Shad, R. Tesch and Y. Y. Ting, *Frontiers in Energy Research*, 2023, **10**, 1–14.

(9) J. C. Garcia, J. Bareño, J. Yan, G. Chen, A. Hauser, J. R. Croy and H. Iddir, *The Journal of Physical Chemistry C*, 2017, **121**, 8290–8299.
